# A systematic framework for identifying prognostic necroptosis-related lncRNAs and verification of lncRNA CRNDE/miR-23b-3p/IDH1 regulatory axis in glioma

**DOI:** 10.18632/aging.205180

**Published:** 2023-11-06

**Authors:** Yangxia Chen, Di Hu, Fang Wang, Cheng Huang, Hesong Xie, Ling Jin

**Affiliations:** 1 Department of Dermatology, The First Affiliated Hospital of Jinan University, Jinan University, Guangzhou, China; 2 Department of Dermatology, Dermatology Hospital, Southern Medical University, Guangzhou, China; 3 Department of Neurology and Stroke Centre, The First Affiliated Hospital of Jinan University, Jinan University, Guangzhou, China; 4 Department of Traditional Chinese Medicine, The First Affiliated Hospital of Jinan University, Jinan University, Guangzhou, China

**Keywords:** glioma, necroptosis, lncRNA CRNDE, miR-23b-3p, IDH1

## Abstract

Glioma remains the most frequent malignancy of the central nervous system. Recently, necroptosis has been identified as a cell death process that mediates the proliferation and development of tumor cells. LncRNAs play a key role in the diagnosis and treatment of various diseases. However, the impact that necrosis-related lncRNAs (NRLs) have on glioma remains unclear. In our studies, we selected 9 NRLs to construct a prognostic model. Meanwhile, we assessed the survival curves of these 9 NRLs. Our findings found ADGRA1-AS1 and WAC-AS1 were protective lncRNAs, while MIR210HG, LINC01503, CRNDE, HOXC-AS1, ZIM2-AS1, MIR22HG and PLBD1-AS1 were risk lncRNAs. Specifically, 12 immune cells, 25 immune-correlated pathways, and TME score were differentially expressed in the both risk groups. Additionally, the study predicted and validated the necroptosis-related lncRNA CRNDE/miR-23b-3p/IDH1 axis. CRNDE was strongly expressed in glioma specimens and several cell lines. Inhibiting CRNDE resulted in a substantial reduction in the proliferation and migration of U-118MG and U251 cells. Furthermore, the study predicted that CRNDE may exhibit oncogenic features by adsorbing miR-23b-3p and positively regulating IDH1 expression. Overall, the study constructed a prognostic model in glioma, and predicted a lncRNA CRNDE/miR-23b-3p/IDH1 axis, which could potentially be useful for gene therapy of glioma.

## INTRODUCTION

Glioma remains the most common type of central nervous system tumor, with highly mortality and morbidity [1]. Despite advancements in research, glioma is insensitive to radiotherapy and chemotherapy, which results a poor prognosis [2]. Although several biomarkers and diagnostic tools were identified over the recent years [3, 4], they remain in the molecular research stage [5]. To uncover the prognostic genetic features of glioma and to receive precise clinical information, it is crucial to pinpoint potential biomarkers and prognostic model.

Necroptosis is a regulated form of cell death that does not rely on the activation of cysteine proteases. It is particularly important in inflammasome-related diseases and compounds [6]. Necroptosis is distinct from other forms of cell death in terms of both morphology and mechanism, with RIPK1/RIPK3/MLKL-mediated pathways being a defining characteristic [7]. In recent years, numerous studies have established a strong association between necroptosis and human cancer. Recent studies have shown that necroptosis may play a role in regulating tumor repopulation in colorectal cancer [8]. Additionally, necroptosis has been found to promote the migration and invasion of pancreatic cancer cells by regulating the CXCL5-CXCR2 axis [9]. These findings suggest that necroptosis may be closely related to the prognostic development of various cancers. However, the role of necroptosis in glioma remains unclear.

LncRNA is a functional non-coding RNA that has recently been discovered to mediate various mechanisms and play vital roles in numerous cancer processes [10, 11]. Recently, it has been demonstrated that lncRNAs regulate the early development of glioma via various signals. Previous studies have illustrated that lncRNA H19 activates the VEGF signaling pathway via sparing miR-138 to promote glioma angiogenesis [12], and that lncRNA BCYRN1 regulates the PTEN/AKT/p21 pathway to inhibit glioma tumorigenesis [13]. Additionally, lncRNA PVT1 has been found to accelerate glioma cell migration, proliferation, and invasion [14]. LncRNA TRINGS could inhibit the necroptosis pathway to protect the cancer cells [15]. While the role of lncRNAs in glioma tumorigenesis has been extensively studied, their impact on necroptosis remains largely unexplored.

The aim of our study was to investigate the expression profiles of NRLs and their correlation with the immune microenvironment. We also aimed to validate and prospect potentially intrinsic molecular regulation in glioma. Our results can serve as a valuable reference for the development of efficient online prognostic biomarkers and practical clinical diagnosis of glioma.

## MATERIALS AND METHODS

### Data collection

Transcriptional data and clinical characteristics of glioma patients, including glioblastoma multiforme (GBM) and low-grade glioma (LGG), were obtained from The Cancer Genome Atlas (TCGA) portal (https://portal.gdc.cancer.gov/).

### Identification of necroptosis-related genes (NRGs)

To identify NRGs, we referred to previous reports [16, 17] and selected 36 mRNAs as DEGs using the ‘limma’ and ‘pheatmap’ packages. These DEGs were used to establish a PPI network using the search tool for the retrieval of interacting genes (STRING, https://string-db.org/) with an interaction score set at 0.9.

### KEGG and GO enrichment analysis

The biological characteristics of the NRGs were analyzed using the GO (http://www.geneontology.org/) and the signaling pathway of NRGs was detected using the KEGG (http://www.genome.jp/kegg/) enrichment analyses.

### Identification of necroptosis-related lncRNAs (NRLs) and prognosis model construction

In this study, the researchers used Pearson correlation analysis to identify lncRNAs related to necroptosis-related DEGs. They then randomly divided the 667 included cases at a 1:1 ratio into training and validation cohorts. NRLs were selected using three different analyses. In order to establish a prognostic model, these NRLs were selected and used to calculate a risk score based on their expression levels and coefficients. The hazard ratio (HR) was then analyzed to differentiate between protective lncRNA (HR>1) and risk lncRNA (HR<1). Finally, the patients were divided into two groups based on their level of risk: high and low.

### PCA and GSEA analysis

To converge glioma patients based on the expression patterns of NRGs, PCA was performed. The distribution of patients was then visualized using 3D scatter plots. To analyze the differences in biological pathways, GSEA was utilized.

### Immunoinfiltration analysis

In this study, we utilized the ‘gsva’ R package to conduct single-sample gene set enrichment analysis (ssGSEA) on immune cells and immune-related pathways. Our analysis aimed to determine the infiltration fractions of immune cells and the activities of immune-related pathways. Additionally, we performed ssGSEA to explore the correlation between the risk model and immune cell infiltration, as well as the correlation between NRLs and immunity.

### Construction of ceRNA network

In order to better understand the potential mechanism of NRLs in glioma, a ceRNA network was constructed. The miRNA targets connecting to NRLs were predicted using Mircode (http://www.mircode.org). Following miRNA identification, TargetScan (http://www.targetscan.org/vert_72/) and miRDB databases (http://mirdb.org/) were used to predict mRNA targets that interact with the miRNAs.

### Cell culture

The glioma cell lines U-118MG (Procell CL-0458) and U251 (Procell CL-0237) used in this study were generously provided by Procell Life Science & Technology Co., Ltd. (Wuhan, China). Glioma cells were cultured in DMEM medium (Gibco, USA). They were stored at 37° C in a humidified incubator with 5% CO_2_.

### RT-qPCR analysis

Trizol (Beyotime, Shanghai, China) was employed to extract the total RNA from U-118MG and U251 cells. RNA was reverse-transcribed into complementary DNA (cDNA) using SuperScript VILO cDNA Kit (Thermo Fisher Scientific, Inc., USA). SYBR Green qPCR Master Mix (Applied Biosystems, USA) was applied to detect the quantitative PCR from the 2^−ΔΔCt^ method. The primers were listed in Table 1.

**Table 1:** Primer list.

**Gene**	**Primers**
CRNDE	Forward: 5'- AAATTCATCCCAAGGCTGGT -3'
	Reverse: 5'- AAACCACTCGAGCACTTTGA -3'
IDH1	Forward: 5'- ATGCAAGGAGATGAAATGACACG -3'
	Reverse: 5'- GCATCACGATTCTCTATGCCTAA -3'
GADPH	Forward: 5'- CCAGGTGGTCTCCTCTGA -3'
	Reverse: 5'- GCTGTAGCCAAATCGTTGT -3'
U6	Forward: 5'- CGCTTCGGCAGCACATATAC -3'
	Reverse: 5'- TTCACGAATTTGCGTGTCATC -3'
miR-23b-3p	Forward: 5'- TCACATTGCCAGGGATTAC -3'
	Reverse: 5'- GAACATGTCTGCGTATCTC -3'

### Statistical analysis

In this study, we used ANOVA and paired samples t-test to evaluate between-group differences. We also used Pearson’s correlation test to analyze the correlation. Statistical analyses were conducted using SPSS 25.0 software and GraphPad Prism 8.0.1. All experiments were independently performed and repeated three times. All values are shown as mean ± SEM, ns indicates no significance, and was considered statistically significant when a significant level of *P*<0.05 occurred.

### Data availability statement

The datasets in this study were downloaded from the TCGA (https://cancergenome.nih.gov/) and CGGA (http://www.cgga.org.cn/).

## RESULTS

### Identification of the expression of NRGs in glioma

**Figure 1:**
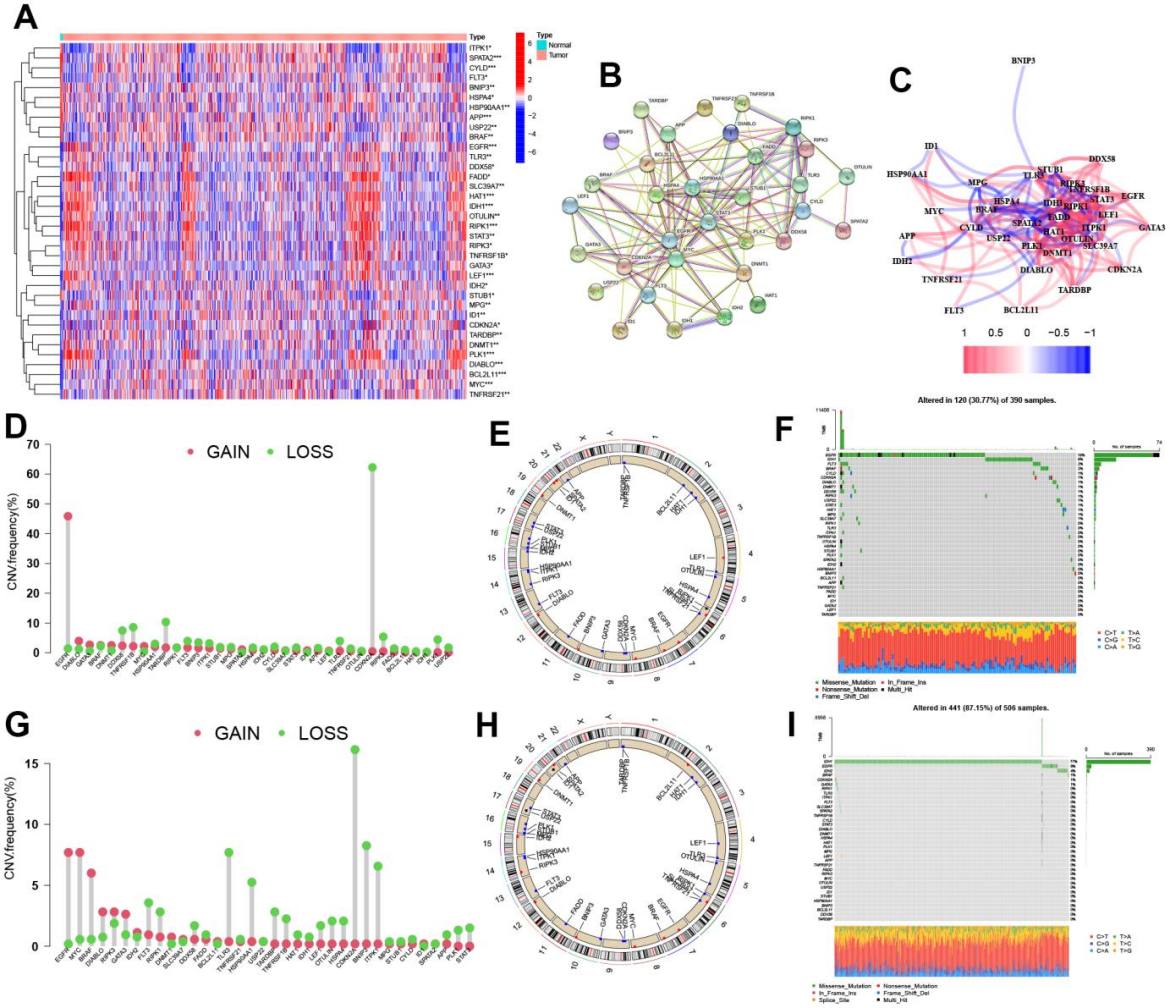
**Identification of the NRGs expression in glioma.** (**A**) Heatmap of 36 NRGs. (**B**, **C**) Network of among 36 NRGs. (**D**) CNV variation frequency of NRGs. (**E**) Location of CNV alterations. (**F**) Genetic alterations of NRGs. (**G**) CNV variation frequency of NRGs. (**H**) Location of CNV alterations. (**I**) Genetic alterations of NRGs. CNV, copy number variation.

The levels of 67 genes related to cell necroptosis were compared between glioma and normal tissues using the TCGA dataset. As a result, 36 NRGs were identified as differentially expressed genes (DEGs). Out of these NRGs, 19 NRGs (EGLN3, HILPDA, HMBS, HYOU1, BNIP3, etc.) were found to be enriched in glioma tissues while 13 genes (SLC4A1, IL6, EPAS1, ACE, HMOX1, etc.) were decreased compared to normal tissues (Figure 1A). Protein-protein interaction (PPI) analysis was conducted among the 36 DEGs, which revealed a highly complex and specific interaction pattern (Figure 1B). This was further confirmed by the relationship network between all NRGs (Figure 1C). The analysis of mutations in 36 NRGs revealed that EGFR had the highest mutation frequency in GBM while IDH1 had the highest mutation frequency in LGG (Figure 1F, 1I). Additionally, a majority of the NRGs showed a trend where the frequency of copy number ‘loss’ was higher than that of ‘gain’, particularly in genes such as CDKN2A, TARDBP, TNFRSF1B, among others (Figure 1D, 1E, 1G, 1H).

### Biological function enrichment study of NRGs

**Figure 2:**
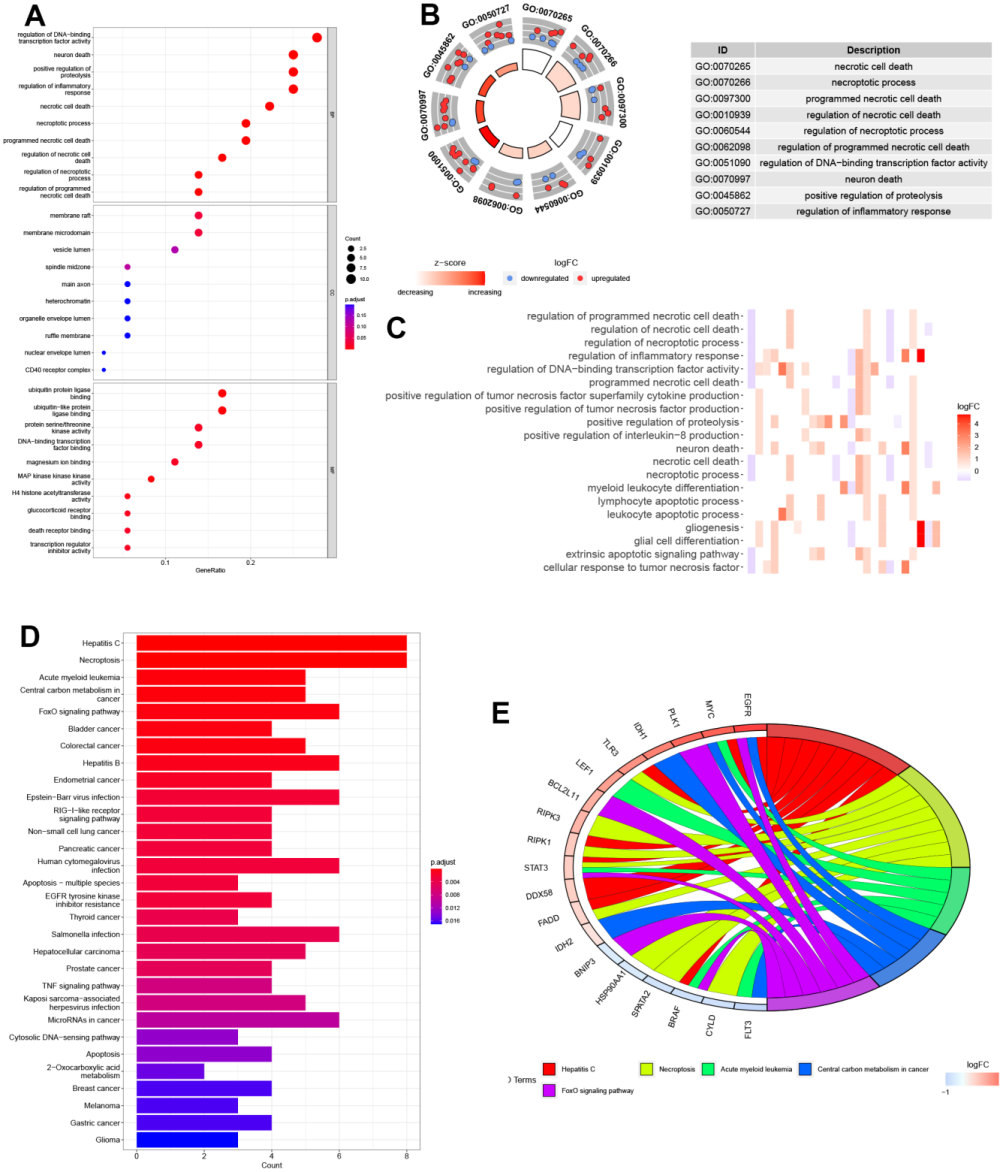
**Biological functional enrichment research of NRGs.** (**A**–**C**) GO enrichment of NRGs. (**D**, **E**) KEGG pathways of NRGs.

We conducted a biological functional enrichment analysis of 36 DEGs related to necroptosis using GO and KEGG databases. The GO analysis revealed that these genes were enriched in biological processes such as ‘regulation of DNA-binding’, ‘neuron death’, ‘regulation of proteolysis’, and ‘regulation of inflammatory response’. In cell component, these genes were enriched in ‘membrane raft’ and ‘membrane microdomain’. The molecular function analysis showed that these genes were associated with ‘ubiquitin protein ligase binding’ and ‘ubiquitin-like protein ligase binding’. The KEGG analysis indicated that these genes were involved in various pathways such as ‘hepatitis C’, ‘necroptosis’, ‘acute myeloid leukemia’, and ‘central carbon metabolism in cancer’ (Figure 2A–2E).

### Identification and co-expression network construction of NRLs

**Figure 3:**
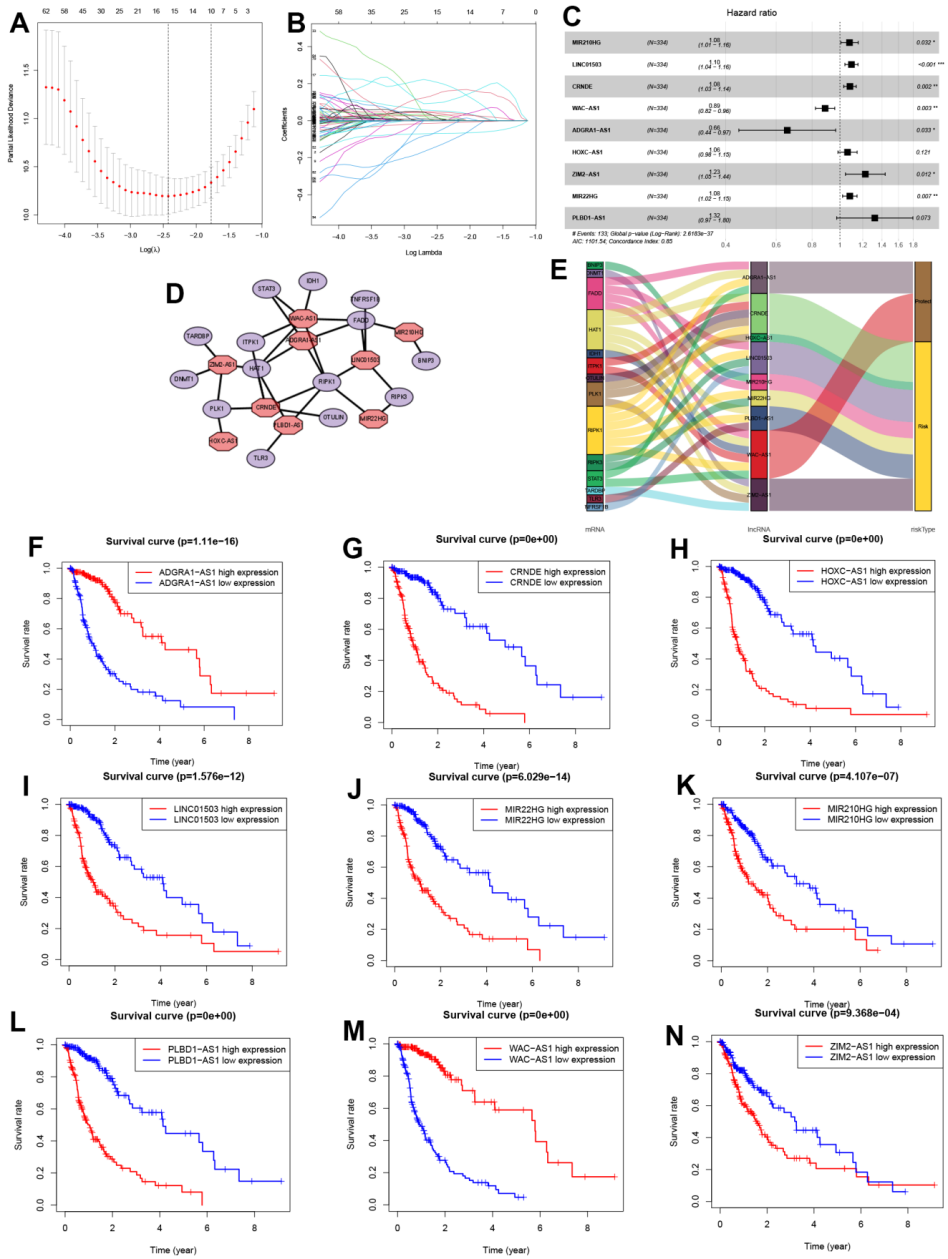
**Identification of NRLs and their subsistence analysis.** (**A**, **B**) LASSO Cox algorithm. (**C**) The risk model of 9 NRLs. (**D**) The co-expression structure. (**E**) Sankey diagram. (**F**–**N**) The Kaplan-Meier analysis of 9 NRLs.

This study involved 667 cases, which were divided into training (n=334) and validation (n=333) cohorts (Figure 3) at a 1:1 ratio. Using the Pearson correlation method, 221 lncRNAs related to 36 NRGs were identified for future analysis (|R|>0.4 and *P*<0.001). Next, the univariate Cox regression analysis and LASSO Cox algorithm were used to reduce multicollinearity, resulting in the identification of 15 NRLs (Figure 3A, 3B). After subsequent multivariate analysis, 9 lncRNAs, including MIR210HG, LINC01503, CRNDE, WAC-AS1, ADGRA1-AS1, HOXC-AS1, ZIM2-AS1, MIR22HG and PLBD1-AS1, were selected to construct the risk model (Figure 3C), with a global *p*-value of 2.6183e-37. These 9 NRLs, ADGRA1-AS1 and WAC-AS1 were identified as 2 protective NRLs, while MIR210HG, LINC01503, CRNDE, HOXC-AS1, ZIM2-AS1, MIR22HG and PLBD1-AS1 were identified as 7 risk NRLs (Figure 3E). According to Figure 3F–3N, the overexpression of 7 NRLs (MIR210HG, LINC01503, CRNDE, HOXC-AS1, ZIM2-AS1, MIR22HG and PLBD1-AS1) was associated with a poor prognosis for glioma patients. However, the opposite was observed for lncRNAs ADGRA1-AS1 and WAC-AS1.

### Construction of predictive risk model in glioma patients

**Figure 4:**
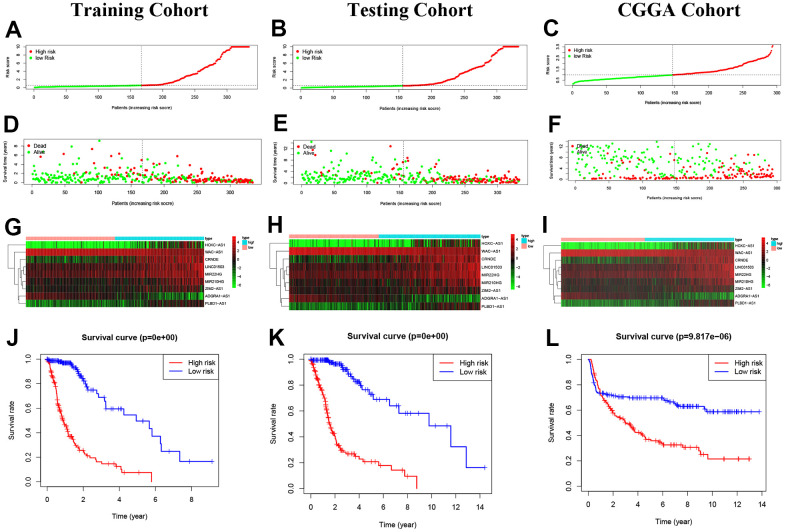
**Construction of predictive risk model in glioma patients.** (**A**–**C**) The two risk groups risk in the three cohorts. (**D**–**F**) The survival status of glioma patients. (**G**–**I**) The heatmap of 9 NRLs. (**J**–**L**) The Kaplan-Meier analysis.

Based on the median risk score of the 9 NRLs with “MIR210HG×(0.076500)+LINC01503×(0.092220)+CRNDE×(0.080224)+WAC-AS1×(-0.119002)+ADRA1-AS1×(-0.422643)+HOXC-AS1×(0.060804)+ZIM2-AS1×(0.203541)+MIR22HG×(0.079291)+PLBD1-AS1×(0.279336)”, 334 glioma patients in the training cohort and glioma patients in CGGA cohort (external validation) as well as 333 in the testing cohort were divided into high- and low-risk groups (Figure 4A–4F). The expression levels of 9 NRLs were analyzed for these three groups and presented in a heatmap (Figure 4G–4I). The results showed that patients in the high-risk group had worse OS compared to those in the low-risk group, as demonstrated by the Kaplan-Meier analysis (Figure 4J–4L).

### Prognosis value of model NRLs in glioma

**Figure 5:**
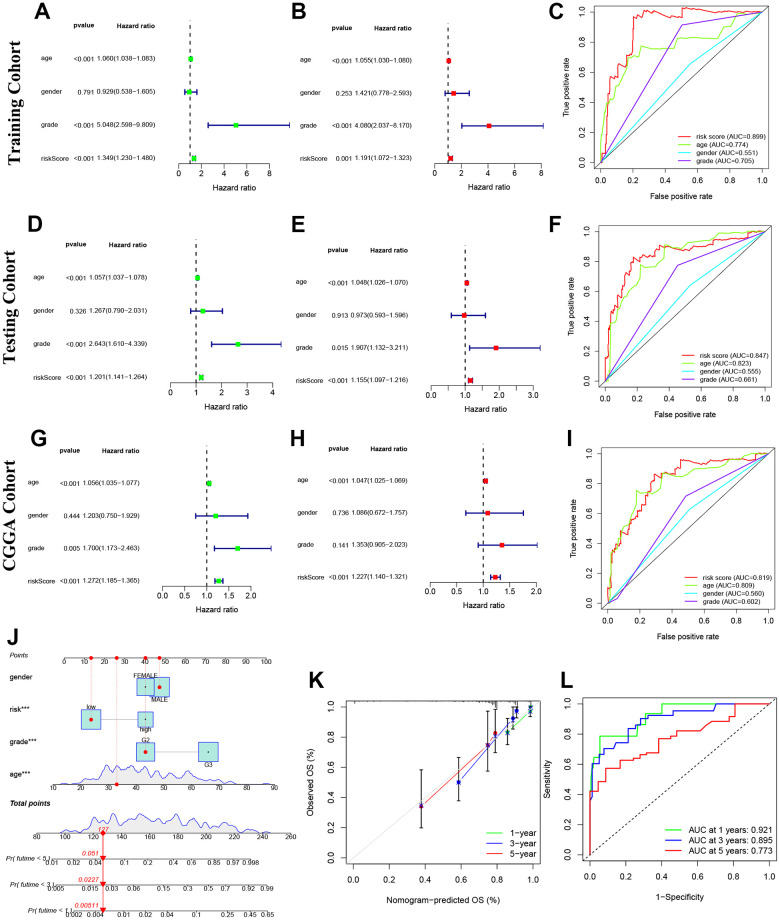
**Prognosis value of model NRLs in glioma.** (**A**–**C**) Univariate analysis, multivariate analysis, and the ROC curves in training cohort. (**D**–**F**) Univariate analysis, multivariate analysis, and the ROC curves in testing cohort. (**G**–**I**) Univariate analysis, multivariate analysis, and the ROC curves in CGGA cohort. (**J**) Nomogram model. (**K**) Calibration curve. (**L**) The ROC curves.

Our study found that the risk model based on NRLs was an independent factor in predicting the OS of glioma patients, compared to other clinical factors (Figure 5A–5I). Cox regression analyses showed that this model had a higher sensitivity and specificity in predicting OS, as confirmed by the higher area under the curve (AUC) values in the ROC curve analysis. Specifically, the AUC values were 0.899, 0.847 and 0.819 in the training, testing and CGGA cohorts, respectively (Figure 5A–5I).

### Construction and detection of a glioma prediction nomogram

Predictive nomograms were constructed and tested to determine the survival value of glioma patients at 1, 3, and 5 years. The independent prognostic factors identified were grade, age, and risk score, as shown in Figure 5J. The calibration curve results indicated that the nomogram model accurately predicted the OS of glioma patients, as shown in Figure 5K. The model also demonstrated high sensitivity and efficacy with AUC values of 0.921, 0.895 and 0.773 for 1-year, 3-year, and 5-year predictions, respectively, as shown in Figure 5L.

### Pathways of the risk model and PCA analyses

**Figure 6:**
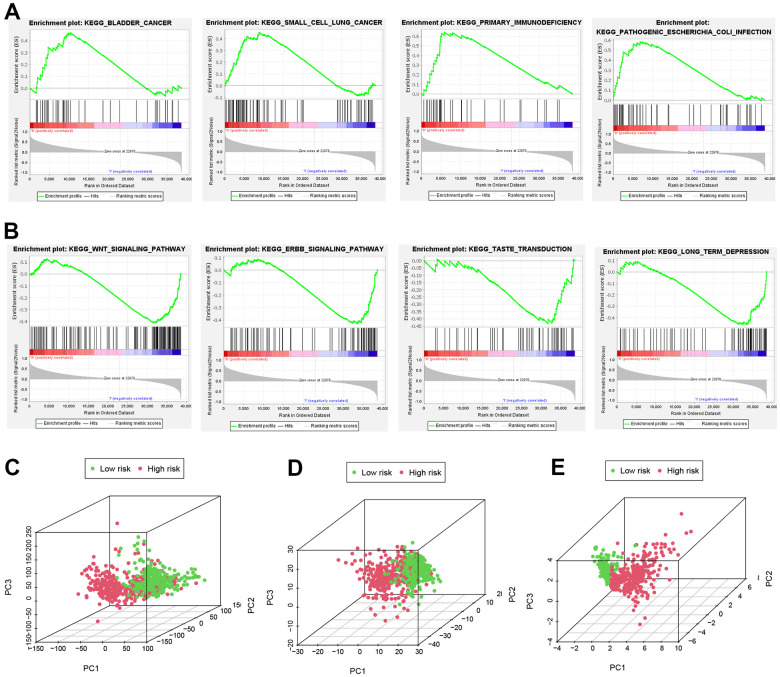
**Important pathways and PCA analyses.** (**A**, **B**) GSEA analysis. (**C**, **D**) All NRGs, lncRNAs and (**E**) NRLs in PCA analysis using 3D scatterplot.

GSEA analysis was conducted to identify important pathways of gene expression between high and low risk groups. The results suggest that genes expressed in the high-risk group are more involved in immune and tumor-related pathways (Figure 6A), while genes expressed in the low-risk group are more inclined to WNT and ERBB signaling pathways (Figure 6B). Additionally, PCA analyses using 3D scatter plots demonstrated different distribution patterns in patients for all NRGs, all NRLs, and 9 NRLs, respectively (Figure 6C–6E). These findings indicate that the risk model developed in this study can effectively distinguish between patients in the high and low risk groups.

### Characteristics of the TME in different groups

**Figure 7:**
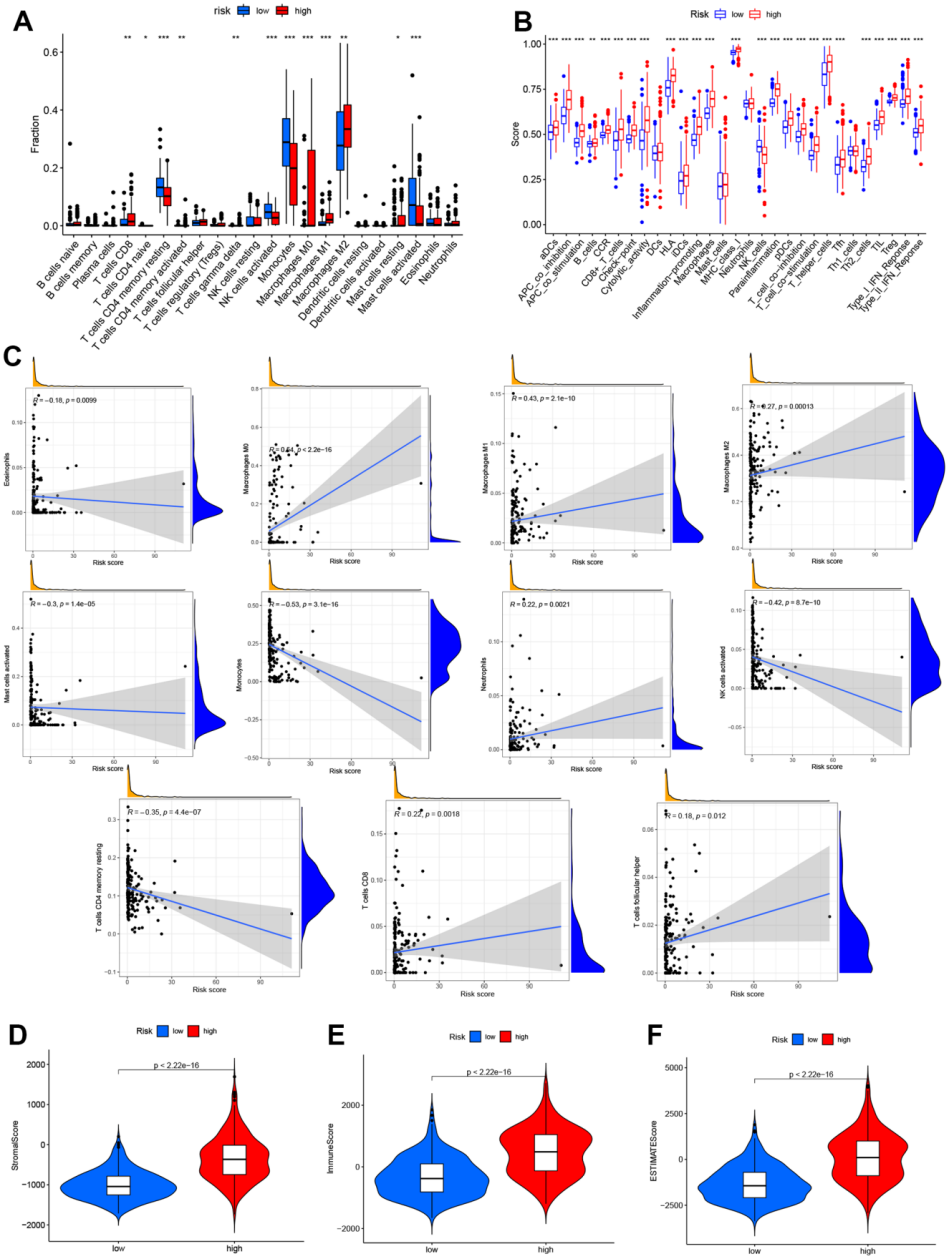
**Analysis of immune activity in different groups.** Comparison of immune cells, (**A**, **B**) various immune-correlated pathways. (**B**, **C**) Immune infiltrations of the risk model. (**D**–**F**) TME score of the two groups.

In order to assess the impact of risk models on glioma TME, we utilized “CIBERSORT” algorithm to analyze 22 immune cell types in glioma. Our findings revealed that 12 immune cell types exhibited different expression in both risk groups (Figure 7A). Furthermore, it was observed that high-risk groups in the TCGA cohort had significantly higher scores in most immune-related pathways compared to low-risk groups (Figure 7B). On the one hand, a positive correlation was found between the survival outcome of glioma patients and a high degree of Macrophages (M0, M1, M2), T-cell CD8, neutrophils and T-cell follicular helpers. On the other hand, there is a negative correlation between survival outcomes in glioma patients and high degrees of eosinophils, mast cell activation, Monocytes, NK cell activation and T cell CD4 memory quiescence. Figure 7C illustrates the association between the risk model and immune cell infiltration. Additionally, the study utilized the ‘TME’ package to investigate the TME scores of both groups. The results showed that stromal and immune cells were more concentrated in the high-risk group (Figure 7D–7F). Thus, these results highlight the higher TME scores in the high group of glioma patients.

### Correlation analysis of 9 NRLs and tumor microenvironment infiltration

**Figure 8:**
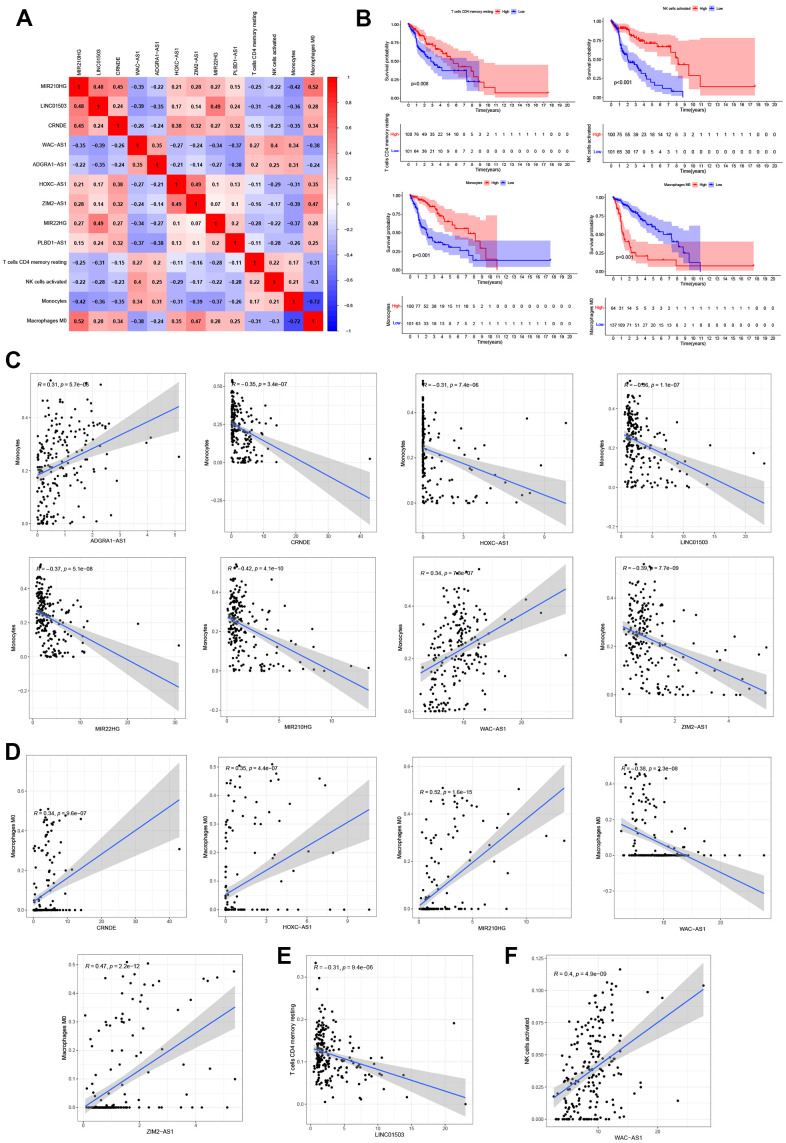
**Correlation analysis of 9 NRLs and immunity.** (**A**) Heatmap of 9 NRLs and 4 immune cells (T cells CD4 memory resting, NK cells activated, Monocytes and Macrophages M0). (**B**) Kaplan-Meier survival analysis. (**C**–**F**) The relationship between 9 NRLs and 4 infiltrations of immune cells.

The infiltration relationships of 9 NRLs with 4 immune cells (T cells CD4 memory resting, NK cells activated, Monocytes and Macrophages M0) were shown in Figure 8A. Then we found that these immune cell infiltrations markedly impacted the survival outcome of glioma patients, high expression levels of 3 immune cells (T-cell CD4 memory resting, NK cell activated and Monocytes) tended to have a better prognosis of glioma patients significantly, however, the opposite resulted in Macrophages M0 (Figure 8B). Furthermore, the relationship between 9 NRLs and immune cell infiltration were depicted in Figure 8C–8F, and it is noted that Monocytes were positively correlated with 2 lncRNAs (ADGRA1-AS1 and WAC-AS1) and with 6 lncRNAs (CRNDE, HOXC-AS1, LINC01503, MIR22HG, MIR210HG and ZIM2-AS1) were negatively correlated (Figure 8C). Thus, there was a positive correlation between Macrophages M0 and four lncRNAs (CRNDE, HOXC-AS1, MIR210HG and ZIM2-AS1), and a negative correlation between Macrophages M0 and lncRNAs WAC-AS1 (Figure 8D). In addition, T cell CD4 memory recovery was negatively correlated with lncRNA LINC01503 (Figure 8E), while NK cell activation was positively correlated with lncRNA WAC-AS1 (Figure 8F). Altogether, these findings highlighted the immunomodulatory role of NRLs.

### Construction of the axis of lncRNA CRNDE/miR-23b-3p/IDH1

**Figure 9:**
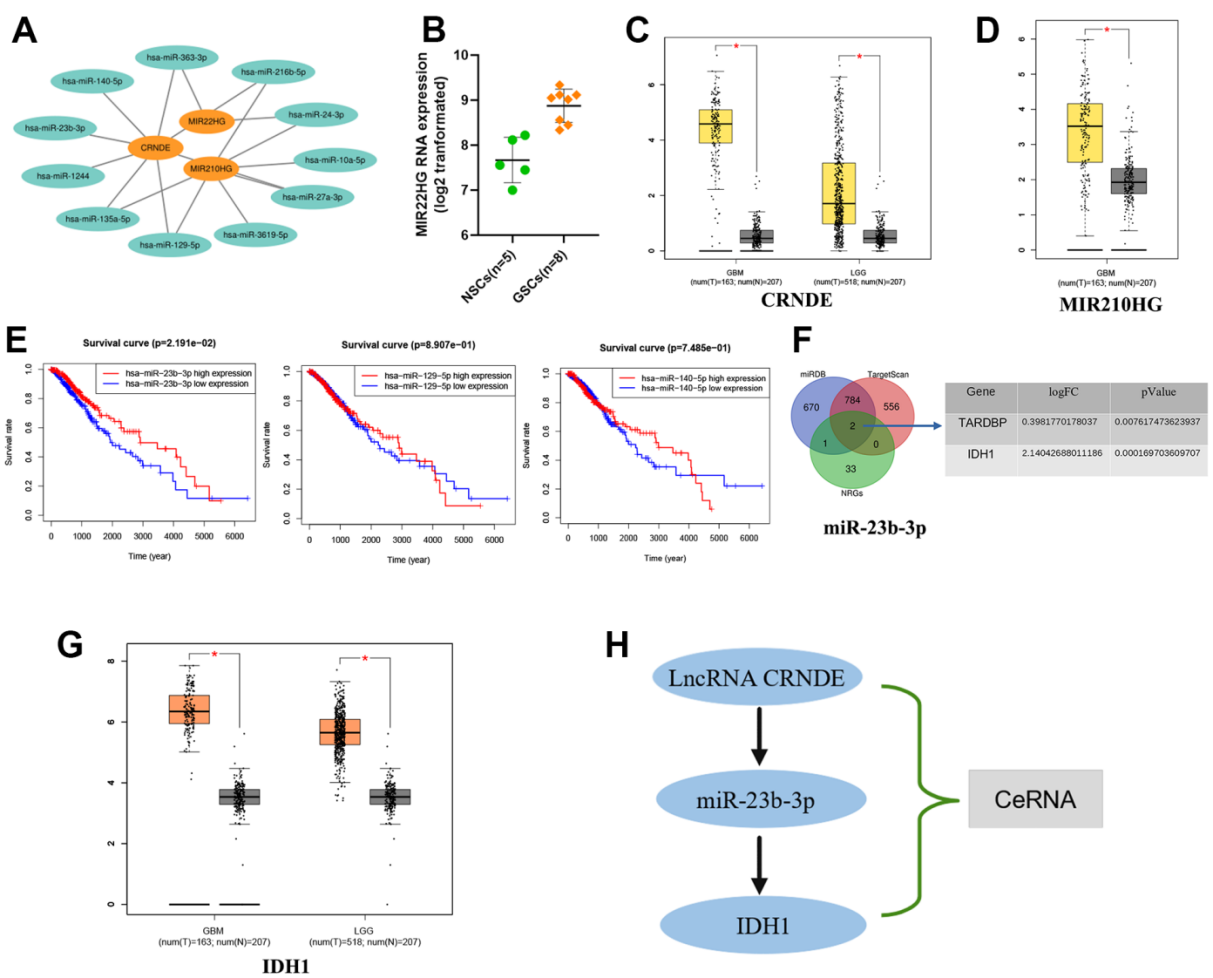
**Construction of the axis of lncRNA-miRNA-mRNA.** (**A**) 3 NRLs (MIR22HG, CRNDE and MIR210HG) bound to various miRNAs. (**B**–**D**) The expression of 3 NRLs. (**E**) Kaplan-Meier survival analysis of 3 miRNAs (miR-23b-3p, miR-129-5p and miR-140-5P). (**F**) Venn diagram. (**G**) The expression of IDH1 by GEPIA database. (**H**) The ceRNA of lncRNA CRNDE/miR-23b-3p/IDH1 axis.

According to Mircode database (Figure 9A), we found that lncRNA MIR22HG bound 2 miRNAs (miR-24-3p, miR-363-3p), lncRNA CRNDE bound 8 miRNAs (miR-135a-5p, miR-140-5p, miR-1244, miR-216b-5p, miR-23b-3p, miR-27a-3p, miR-129-5p and miR-363-3p) and lncRNA MIR210HG binded 7 miRNAs (miR-135a-5p, miR-3619-5p, miR-216b-5p, miR-24-3p, miR-27a-3p, miR-10a-5p and miR-129-5p using ‘Perl’ software (Figure 9A). Among these miRNAs, 5 miRNAs (miR-363-3p, miR-140-5p, miR-23b-3p, miR-129-5p and miR-135a-5p) were reported to be lowly expressed in glioma [18–22], which were contrary to the expression of the target 3 lncRNAs (Figure 9B–9D). However only 3 miRNAs (miR-23b-3p, miR-129-5p and miR-140-5p) could affect the patients’ OS significantly, which identified as targets (Figure 9E). Based on this result, we then found 2 NRGs (TARDBP and IDH1), as the downstream targets of miR-23b-3p (Figure 9F). Moreover, we revel that only IDH1 was up-regulated expression in glioma tissues compared to normal by GEPIA (http://gepia.cancer-pku.cn/) database (Figure 9G). Thus, the ceRNA of lncRNA CRNDE/miR-23b-3p/IDH1 axis was formed, which may play an integral role in glioma progression (Figure 9H).

### The expression of lncRNA CRNDE, IDH1 and miR-23b-3p and the function of lncRNA CRNDE in glioma cell lines

**Figure 10:**
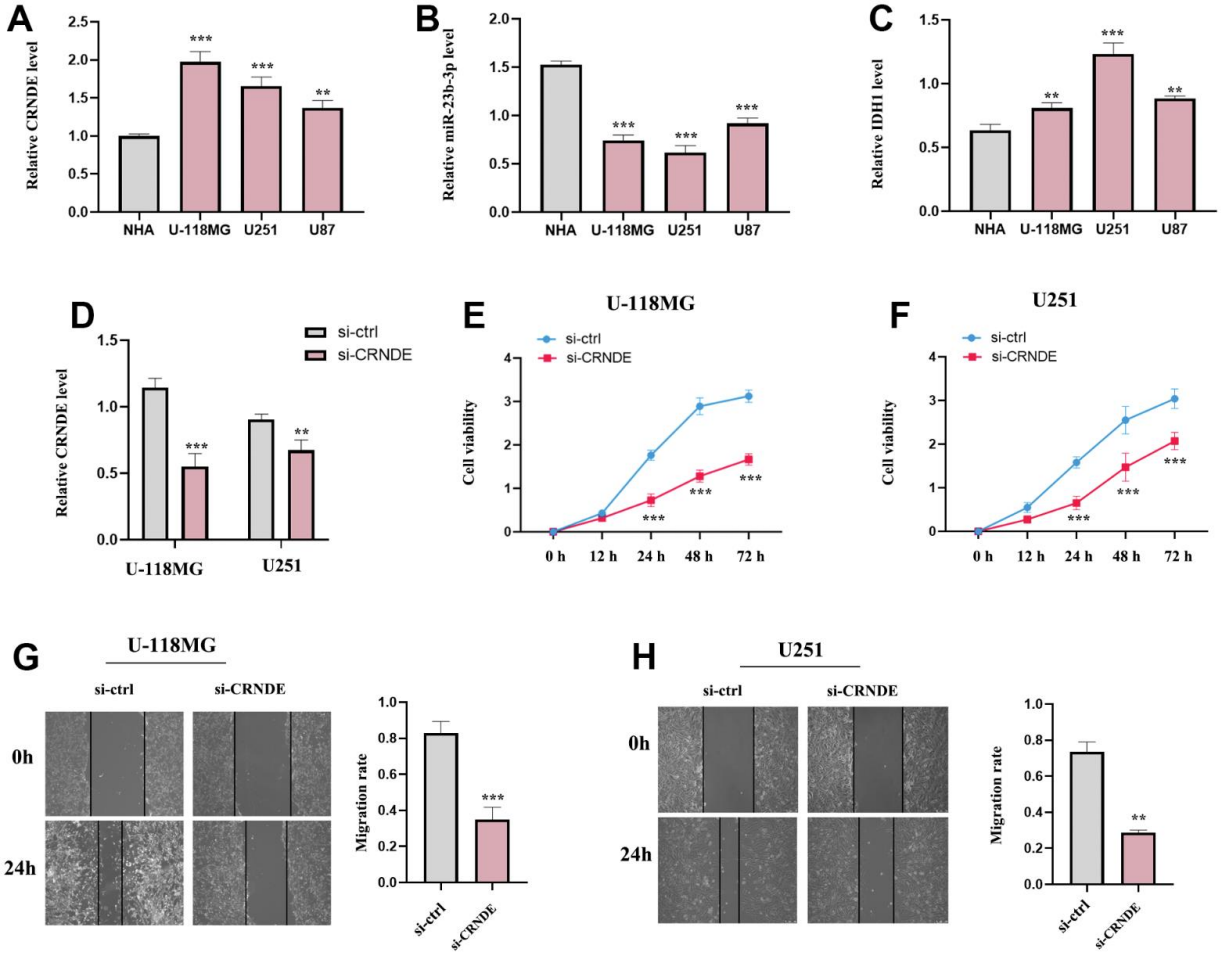
**The expression of lncRNA CRNDE, IDH1 and miR-23b-3p and the function of lncRNA CRNDE in glioma cell lines.** (**A**) LncRNA CRNDE expression in different glioma cell lines (U-118MG, U251 and U87) compared to normal astrocytes (NHA) were investigated by qRT-PCR. (**B**) miR-23b-3p expression in different glioma cell lines (U-118MG, U251 and U87) compared to normal astrocytes (NHA) were estimated by qRT-PCR. (**C**) IDH1 expression in different glioma cell lines (U-118MG, U251 and U87) compared to normal astrocytes (NHA) were estimated by qRT-PCR. (**D**) The efficiency of si-CRNDE. (**E**, **F**) CCK-8 assays in U-118MG and U251 cells. (**G**, **H**) Wound healing assays in U-118MG and U251 cells. *p<0.05; **p<0.01; ***p<0.001.

Then, we selected several glioma cell lines (U-118MG, U251 and U87) for experimental validation *in vitro*, normal astrocytes (NHA) as the control group (Figure 10A–10C). and the result of RT-qPCR suggested that the levels of lncRNA CRNDE and IDHI were increased while miR-23b-3p were decreased in the 3 cell lines (U-118MG, U251 and U87) relative to NHA (Figure 10A–10C). Next, we detected the subtle effect of lncRNA CRNDE on the aggressive capacities of glioma *in vitro*. Our results revealed that the ectopic down-expression of CRNDE in U-118MG and U251 cells markedly inhibited glioma cell proliferation and migration capabilities (Figure 10D–10H) as evidenced by CCK-8 and wound healing assays. These aforementioned results identified a critical role of lncRNA CRNDE in promoting glioma metastasis.

## DISCUSSION

In this study, we have systematically identified NRLs based on the previous involvement of lncRNAs and NRGs in glioma, and in parallel, we constructed unique risk models with various characteristics to predict the prognosis of glioma patients. Specifically, we firstly compared all NRGs in glioma and normal tissues, and finally identified 36 NRGs with differential expression called “DEGs”. Secondly, we developed a risk model consisting of 9 NRLs to generate two risk groups of glioma patients. Again, by comparing with other clinical factors, the risk score owns high sensitivity and specificity. Subsequently, we analyzed the immune cell infiltration and risk models and magically identified an immunomodulatory role of NRLs in both risk groups. Finally, we concluded by making predictions and speculating that the CRNDE/miR-23b-3p/IDH1 network may potentially play a role in the advancement of glioma.

It has been established in many previous studies that lncRNAs can play an essential role in some aspects of necrosis in different malignancies, for instance, Harari-Steinfeld et al. discovered that lncRNA H19-derived miR-675 could stimulate hepatic necrosis through regulating FADD [23]. Wang et al. identified that lncRNA necrosis-related factor (NRF) can regulate cardiomyocyte necrosis via the RIPK1/RIPK3 axis [24]. LncRNA TRINGS can blind STRAP and control the STRAP-GSK3β-NF-κB necrotic pathway to rescue tumor cells. Less wonderfully, however, studies on NRLs in gliomas, particularly their potential capacity to make predictions for glioma patients, are significantly underdeveloped. Thus, we developed a predictive model based on 9 NRLs, including MIR210HG, LINC01503, CRNDE, WAC-AS1, ADGRA1-AS1, HOXC-AS1, ZIM2-AS1, MIR22HG, and PLBD1-AS1. Interestingly, some of these lncRNAs were already reported to be engaged in the pathogenesis of a number of tumor diseases. As an example, lncRNA MIR210HG was revealed to significantly accelerate the aggregation of glioblastoma by Ho et al. [25]. LncRNA MIR210HG was also reported to function as a biomarker in the prognosis of hepatocellular carcinoma according to Wang et al. [26]. Moreover, lncRNA LINC01503 was also reported to be involved in diseases such as cervical cancer [27] and ovarian cancer [28]. Besides that, lncRNA CRNDE has been identified as a risk factor for high invasiveness in glioma patients [29]. Interestingly, lncRNA WAC-AS1 was shown to facilitate the tumor progression of hepatocellular carcinoma [30], contrary to what we found in the present study. LncRNA HOXC-AS1 was revealed to be a prognostic marker and a risk effector for prostate cancer in Takayama’s study [31]. Notably, the long non-coding RNA MIR22HG which was reported to facilitate the progression of glioblastoma via regulation of Wnt/β-catenin signaling [32] coincides with our findings. Similarly, the lncRNAs MIR210HG, LINC01503, CRNDE, HOXC-AS1 and MIR22HG were found to play an essential role as risk factors in prolonging the survival time of glioma patients also in high agreement with our findings.

The prognosis of cancer patients has been observed to be strongly associated with immunotherapy, and the unfolding of the positive side of immunotherapy typically relies on the dynamic regulation of the tumor cells and immunomodulators in the TME [33]. It follows that the exploration of the TME is essential for its effectiveness in not only uncovering new therapeutic strategies but also identifying additional [34, 35]. In our findings, we were able to detect that immune-related infiltrating cells, TME scores, along with immune-related pathways were mainly concentrated in the high-risk group, which also implies that immunosuppressive therapy may be more effective for glioma patients in the high-risk group.

It is believed that lncRNAs are considered as ceRNAs that can act as sponges for miRNA loci subsequently influencing and modulating the biological activity of downstream mRNAs [36, 37]. As an example, the lncRNA-CDC6 can facilitate breast cancer progression by modulating the microRNA-215/CDC6 axis [38]. HOXD-AS1 can act as a ceRNA to enhance the metastasis of liver cancer [39]. LncRNA MT1JP could mediate miR-92a-3p/FBXW7 [40]. From these results, we can easily see that these axes have been widely reported in a variety of diseases, nevertheless, the role of lncRNA-related ceRNAs with glioma is not clearly reflected. Consequently, we constructed a new lncRNA-miRNA-mRNA network and predicted the regulatory axis of lncRNA CRNDE/miR-23b-3p/IDH1 by using biological tools.

According to extensive studies, lncRNA CRNDE also serves as an essential role in ovarian and cervical cancers. LncRNA CRNDE not only stimulates ovarian cancer progression by sponging miR-423-5p to upregulate FSCN1 expression [41], but also activates the miR-183/CCNB1 axis as an oncogene [42]. Thus, we delved into and elaborated the lncRNA CRNDE and IDH1 overexpression and miR-23b-3p expression downregulation in human glioma cells (U-118MG, U251 and U87), which is in line with the previous reports. At the same time, our experimental results also confirmed down-expression of CRNDE in U-118MG and U251 cells markedly inhibited glioma cell proliferation and migration capabilities. In this work, we also predicted that lncRNA CRNDE may interact with miR-23b-3p based on hints from the Mircode database. Based on this prediction, we further explored the downstream mRNA targets of miR-23b-3p, and finally successfully mined the miRDB and TargetScan databases to identify the NRG (IDH1) as precisely the downstream target of miR-23b-3p. Based on these results, we speculated that lncRNA CRNDE played a role as an oncogene in glioma, and might through targeting miR-23b-3p regulating IDH1, which is the focus of our future work.

## CONCLUSIONS

To summarize, we identified nine NRLs including MIR210HG, LINC01503, CRNDE, WAC-AS1, ADGRA1-AS1, HOXC-AS1, ZIM2-AS1, MIR22HG, and PLBD1-AS1. We found a potential network between lncRNA CRNDE, miR-23b-3p, and IDH1, and our *in vivo* experiments partially confirmed the role of lncRNA CRNDE in promoting glioma metastasis through the regulation of miR-23b-3p/IDH1. However, further studies are needed to provide concrete evidence.
